# The Complete Mitochondrial Genome of *Callicarpa americana* L. Reveals the Structural Evolution and Size Differences in Lamiaceae

**DOI:** 10.3390/biology14121747

**Published:** 2025-12-05

**Authors:** Yang Wu, Jiayue Xu, Tenglong Hong, Jing He, Yuxiang Chen, Ye Zhang, Xinyu Hu, Huimin Sun, Li He, Dingkun Liu

**Affiliations:** 1 School of Life Sciences, Jinggangshan University, Ji’an 343009, China; jgsdxzwzy@hotmail.com (Y.W.);; 2 Key Laboratory of Jiangxi Province for Biological Invasion and Biosecurity, School of Life Sciences, Jinggangshan University, Ji’an 343009, China

**Keywords:** American beautyberry, mitochondrial genome, phylogenetic analysis, Lamiaceae

## Abstract

*Callicarpa americana* L. is a member of the Lamiaceae family with important ornamental and medicinal value. Although the chloroplast genome of Lamiaceae has been extensively studied, its mitochondrial genome remains unreported, limiting a comprehensive understanding of the phylogeny and genome evolution of Lamiaceae. In this study, the complete mitochondrial genome of *C. americana* was successfully assembled for the first time. The genome is 499,565 bp in length, showing a complex multi-branched closed-loop structure that contains 37 protein-coding genes, 23 tRNA genes, and 4 rRNA genes. The difference in mitochondrial genome size is relatively large compared to Orobanchaceae species, but the difference in GC content is not obvious. The expansion of genome size was mainly due to the accumulation of non-coding regions and repetitive sequences. Meanwhile, two pairs of long repetitive sequences (LR3 and LR5) mediated homologous recombination. The mitogenome was also identified; there were a total of 494 C-to-U RNA editing sites in protein-coding genes. In addition, 42 mitochondrial plastid DNA fragments (MTPTs) were detected, with a total length of 21,464 bp, accounting for 4.30% of the genome. Repeat sequence analysis showed that tetranucleotide SSR was the most abundant repeat type in the mitochondria of Lamiaceae. Phylogenetic analysis based on the alignment of 32 protein-coding gene sequences showed that *Callicarpa* is sister to the other eight species of Lamiaceae. This work fills an important gap by presenting the first complete mitochondrial genome of *C. americana*, providing an important data resource for further understanding the structural evolution, dynamic recombination mechanism, and phylogeny of the mitochondrial genome of Lamiaceae.

## Simple Summary

Plants in the mint family play important roles in medicine and landscaping, yet very little is known about how their mitochondrial genomes are built and how they change over time. The mitochondrial genome is the part of the cell that helps produce energy, and understanding its structure can provide valuable clues about plant evolution. In this study, we described the complete mitochondrial genome of *Callicarpa americana* L. for the first time. We found that the genome is large, has an unusual branching shape, and contains 64 genes. Many repeated DNA segments were present, and these repeated segments were identified as the main reason why the genome is so large. We also found many places where the genetic code is changed after it is copied, as well as pieces of DNA that originally came from the plant’s chloroplast, the structure responsible for photosynthesis. When we compared this genome to those of other members of the mint family, we found that *Callicarpa* L. forms its own distinct branch in the phylogenetic tree. These findings provide new information that fills a major knowledge gap and will support future work on plant evolution, conservation, and the development of useful plant resources.

## 1. Introduction

*Callicarpa americana* belongs to the family Lamiaceae and is widely distributed across the southeastern United States [1]. The genus *Callicarpa* was first established by Linnaeus in 1753. Approximately 150–190 species of *Callicarpa* are recognized worldwide, primarily distributed in eastern and southeastern Asia [2]. In China, 46 species have been recorded, 31 of which are endemic and grow in forests or shrublands at elevations of 200–2300 m [3]. *Callicarpa americana* commonly grows at forest edges, in shrubs and in open woodlands. After flowering in summer, the plant produces bright purple berries in autumn, attracting numerous birds and other wildlife [1,4]. However, its familial placement was initially uncertain due to high morphological similarity and ambiguous interspecific boundaries, leading to long-standing taxonomic controversy [5]. Especially when subspecies, species, and interspecific variation are difficult to clearly define, the limitations of traditional classification methods become apparent. Traditionally, *Callicarpa* was attributed to Verbenaceae based on morphological traits, but molecular evidence has since reassigned it to Lamiaceae [6]. Members of this family are valued for their vivid fruit colors and spherical, lustrous berries, making them widely used as ornamental plants in landscaping and ecological restoration. Beyond their ornamental function, the roots, branches, and leaves of several *Callicarpa* species possess pharmacological properties, including hemostatic and anti-inflammatory activities [7]. Recent studies have reported that leaf extracts from these species exhibit mosquito-repellent activity and inhibitory effects against *Propionibacterium acnes* [8], further highlighting their ecological and medicinal value.

Lamiaceae comprises approximately 236 genera and over 7000 species distributed across temperate to tropical regions [6,9]. Many species, such as *Mentha canadensis* L. and *Salvia miltiorrhiza* Bunge, have considerable economic and medicinal value as aromatic and therapeutic plants [10,11]. With the rapid development of high-throughput sequencing, numerous chloroplast genomes from Lamiaceae have been sequenced and analyzed, significantly advancing phylogenetic reconstruction and taxonomic revision of the family [9]. However, in contrast to chloroplast genomes, mitochondrial genome studies remain limited. To date, only a few Lamiaceae species, including *Leonurus japonicus* Houtt. [12], *Mentha spicata* L. [13], *Prunella vulgaris* L. [14], and *S. miltiorrhiza* [15], have had their mitochondrial genomes assembled and characterized. As a result, our understanding of structural evolution, gene content conservation, and the phylogenetic relevance of mitochondrial genomes within Lamiaceae remains limited.

Recent progress in plant mitochondrial genomics has greatly accelerated our understanding of organellar genome architecture and evolution. The advent of long-read sequencing technologies such as PacBio HiFi and Oxford Nanopore has enabled the assembly of high-quality plant mitochondrial genomes in several species, including *L. japonicus* [12] and *Lavandula angustifolia* Mill. [16]. These advances have provided valuable insights into plant mitochondrial genome evolution, RNA editing, and inter-organellar DNA transfer. Plant mitochondrial genomes differ substantially from those of animals; they are typically much larger (100–200 kb on average) and show remarkable variability both between and within species [16,17]. One notable evolutionary feature of plant mitochondria is mitochondrial-to-plastid DNA transfer (MTPT), in which plastid DNA fragments are integrated into the mitochondrial genome [18]. Such events are common in angiosperms and contribute to genome expansion and structural diversity. In addition, extensive RNA editing—primarily C-to-U conversions—further increases transcriptional and functional complexity [17].

To obtain the complete mitochondrial genome of *C. americana*. Long-read PacBio sequencing data were obtained from the National Center for Biotechnology Information (NCBI) and are publicly accessible under BioProject accession number PRJNA529675 (SRR8932628, SRR8932629), as reported by Hamilton et al. [7]. This study reports the first high-quality chromosome-level genome of *C. americana*. The key terpenoid synthase *CamTPS2* was identified, and the genetic basis of its genome evolution and chemical diversity was revealed. These findings provide important resources for the development of natural pesticides and for comparative genomics within Lamiaceae. However, there is a shortage of Lamiaceae mitochondrial genome data in public databases.

To address the absence of mitochondrial genomic data for *Callicarpa*, the complete mitogenome of *C. americana* was assembled and annotated based on the published data. The aim of this research was to characterize the genome structure, repeat composition, RNA editing profile, and MTPT insertions. In addition, comparative and phylogenetic analyses with other Lamiaceae species were conducted to clarify the evolutionary placement of *Callicarpa* and improve understanding of mitogenome evolution within the family.

## 2. Materials and Methods

### 2.1. Sequencing Data Retrieving

The PacBio CLR sequencing reads were acquired from the NCBI SRA database (SRR8932628, SRR8932629). Sequencing was performed using the PacBio Sequel II platform (Pacific Biosciences, Menlo Park, CA, USA) according to the manufacturer’s instructions. PMAT software was then used to assemble the GFA files, and sequences with low coverage or not belonging to the mitochondrial genome were manually removed. The PacBio reads were error-corrected with Canu v2.2 [19], while the short reads were processed for adapter removal and quality trimming with fastp v0.23.4 [20].

### 2.2. Mitochondrial Genome Assembly and Gene Annotation

The PacBio sequencing reads were assembled into mitochondrial genome contigs using PMAT v1.5.3 [21] under the autoMito configuration. In this process, long reads were segmented into ~20 kb fragments via the ‘break_long_reads.py’ script and subsequently assembled with Newbler. Candidate mitochondrial seed contigs were identified through BLASTn v2.13.0 searches [22] against a local database of 24 conserved mitochondrial protein-coding genes (PCGs). Suitable seed sequences were selected for extension using ‘seeds_extension.py’, and mitochondrial fragments were subsequently connected based on their assembly graph. Non-mitochondrial sequences were excluded, and the final assembly graph was examined with Bandage v0.9.0 [23]. Assembly errors were further corrected by short-read alignment applying Pilon v1.24 [24]. Gene annotation was performed using the IPMGA platform (http://www.1kmpg.cn/ipmga/, accessed on 11 June 2024) and refined manually in Geneious v11.1.5 [25]. A graphical representation of the mitogenome was generated with OGDRAW v1.3.1 [26].

### 2.3. Repeat-Mediated Homologous Recombination Analysis

To investigate homologous recombination mediated by repetitive sequences, we extracted sequence fragments containing repeat units and flanking regions exceeding 5000 bp. Long-read mapping to these sequences was performed using BWA-mem v0.7.8-r455 [27]. Read-mapping statistics were calculated using SAMtools v1.3.1 [28]. Mappings were retained only when reads spanned at least 50 bp of flanking sequence on both sides of the repeat. Recombinant structures were annotated based on mapping counts for subsequent analysis.

### 2.4. Identification of Repeat Elements, Codon Usage Bias, and RNA Editing Events

Simple sequence repeats (SSRs) were detected using the MISA web tool (https://webblast.ipk-gatersleben.de/misa/, accessed on 25 August 2020), using minimum repeat thresholds of 10, 5, 4, 3, 3, and 3 for mono-, di-, tri-, tetra-, penta-, and hexanucleotide motifs, respectively [29]. Tandem repeats were identified via the Tandem Repeats Finder v4.09 server under default conditions [30]. Dispersed repeats were characterized using REPuter [31], with parameters set to a Hamming distance of 3, a minimum repeat length of 30 bp, and a maximum of 5000 repeats. Statistical summaries, including repeat class frequency, length distribution, and abundance comparisons, were computed using Python v3.13 scripts to quantify repeat variation across categories. Codon usage patterns and relative synonymous codon usage (RSCU) values were computed in CodonW v1.4.4 [32], and codon bias indices (including ENC and CAI) were statistically summarized to evaluate codon preference. RNA editing sites within mitochondrial protein-coding sequences were predicted using Deepred-Mt (https://github.com/aedera/deepredmt, accessed on 19 July 2024) [33], applying a probability cutoff of ≥0.9, followed by statistical assessment of editing efficiency, editing-site distribution among genes, and category-wise comparisons of edited vs. unedited codons.

### 2.5. Analysis of Mitochondrial Plastid-Derived DNA Fragments and Genome Synteny

The chloroplast genome of *C. americana* (Accession number: MN883825) was obtained from the NCBI’s Genbank database [34]. Homologous regions shared between the mitochondrial and chloroplast genomes were detected through BLASTn v2.13.0 searches [22] (threshold e-value ≤ 1 × 10^−5^) and visualized using Circoletto [35]. Multiple mitogenome alignments from representative Lamiaceae species were conducted by AliTV v1.0.6 [36] (*Ajuga decumbens* PV972819, *Ajuga reptans* NC023103, *Lavandula angustifolia* NC082307, *Rotheca serrata* NC049064, *Salvia rosmarinus* PP992923, *Scutellaria barbata* NC065025, *Scutellaria tsinyunensis* MW553042, *Vitex trifolia* NC065806) and visualized via AliTV’s online interface, filtering sequences shorter than 500 bp. Dispersed repeats exceeding 400 bp were annotated.

### 2.6. Phylogenetic Analysis

Mitogenomes from representative species within Lamiaceae and outgroups were obtained from GenBank; accession numbers for the sequences are provided in Table S1. Protein-coding sequences were retrieved using PhyloSuite v1.2.3 [37]; repetitive sequences were excluded, leaving 32 mitochondrial PCG sequences that were aligned using MAFFT v7.49 [38]. Alignment trimming was performed using the ‘automated1’ option in trimAl v1.2 [39]. To rebuild the phylogenetic relationships, IQ-TREE v2.0.3 was used for series comparison analysis [40], and Model Finder was used for optimal alternative model selection and ultra-fast bootstrap analysis (UF-Boot = 1000) to estimate node support.

## 3. Results

### 3.1. Mitochondrial Genome Assembly

**Figure 1:**
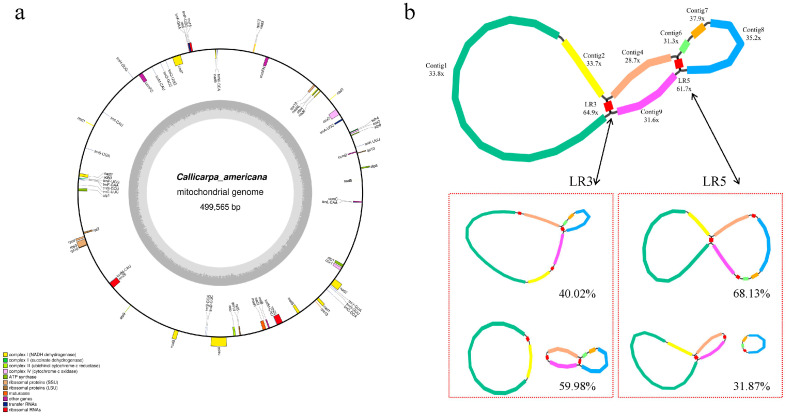
(**a**) Mitochondrial genome annotation and assembly maps of *C*. *americana*. Mitochondrial genome map. The genes belonging to different functional groups are all color-coded cysteines. (**b**) Mitochondrial genome assembly map of *C*. *americana*. Each colored segment is labeled with its coverage depth and ordered by size. The adjacency relationships among fragments were supported by long-read sequencing data, and the possible structure and proportion formed by two long repeat-mediated rearrangements were plotted.

The resulting contigs corresponded to the mitochondrial genome structure of *C*. *americana* (Accession number: PX121882). Contigs were numbered according to length (Figure 1a, Table 1).

The mitochondrial genome of *C. americana* exhibits a complex multi-branched structure; however, it remains a closed-loop structure with an average coverage depth of 111.4x. The mitochondrial genome of *C. americana* consists of seven contigs (contig1–2, contig4, and contig6–9), ranging in length from 7749 bp to 247,270 bp, and two pairs of long repeat sequences (LR3 and LR5), ranging in length from 2164 bp to 4932 bp (Figure 1b, Table 1). The mitochondrial genome of *C. americana* contains 37 protein-coding genes, 23 tRNA genes, and 4 rRNA genes (Table 2, Table S1). The core protein-coding genes include five ATP synthase genes (*atp1*, *atp4*, *atp6*, *atp8*, *atp9*), nine NADH dehydrogenase genes (*nad1*, *nad2*, *nad3*, *nad4*, *nad4L*, *nad5*, *nad6*, *nad7*, *nad9*), four cytochrome C biosynthesis factor genes (*ccmB*, *ccmC*, *ccmFC*, *ccmFN*), three cytochrome oxidase genes (*cox1*, *cox2*, *cox3*), and the key genes *mttB*, *matR*, and *cob*. Non-core genes included four large subunit ribosomal protein genes (*rpl10*, *rpl2*, *rpl5*, *rpl16*), six small subunit ribosomal protein genes (*rps10*, *rps12*, *rps13*, *rps14*, *rps3*, *rps4*), and two succinate dehydrogenase genes (*sdh3*, *sdh4*).

### 3.2. Mitochondrial Genome Homologous Recombination Mediated by Repetitive Sequences

In the *C. americana* mitochondrial genome, a similar proportion of mitochondrial genome recombination mediated by two pairs of long repeat sequences was identified, which were 40.02% (Contig1-LR3-Contig4 and Contig2-LR3-Contig9) and 59.98% (Contig1-LR2-Contig3 and Contig3-LR4-Contig9) of LR3, respectively, and 68.13% (Contig4-LR5-Contig8 and Contig6-LR5-Contig9) and 31.87% (Contig4-LR5-Contig9 and Contig6-LR5-Contig8) of LR5, respectively (Figure 1b, Table 3).

In order to facilitate a description based on the obtained mitochondrial genome recombination ratio, the mitochondrial genome of *C. americana* was processed into a linear molecule with a length of 499,565 bp. The sequence was Contig8-Contig7-Contig6-LR5-Contig9-LR3-Contig2-Contig1-LR3-Contig4-LR5 (Figure 1b). It should be emphasized that this approach is not unique, as the structure of plant mitochondrial DNA is affected by dynamic transformation involving repetitive sequences. This approach was chosen to facilitate subsequent analysis.

### 3.3. Repetitive Sequence Analysis

The number of SSRs detected in the mitochondria of nine Lamiaceae species ranged from 71 to 125 (Table S2). Tetranucleotide repeats were the most abundant, accounting for 35.89% of the total SSRs. Dinucleotide repeats accounted for 15.35%, whereas single nucleotide repeats accounted for 11.26% (Table S2). Hexanucleotide repeats were found in some mitochondrial genomes of Lamiaceae, including three in *Rotheca serrata*, five in *Salvia rosmarinus*, and three in *C. americana* (Table S2). A range of 4 to 12 tandem repeats was detected in the mitochondrial genomes of the nine Lamiaceae species; the repeats ranged from 212 bp to 580 bp (Table S3).

**Figure 2:**
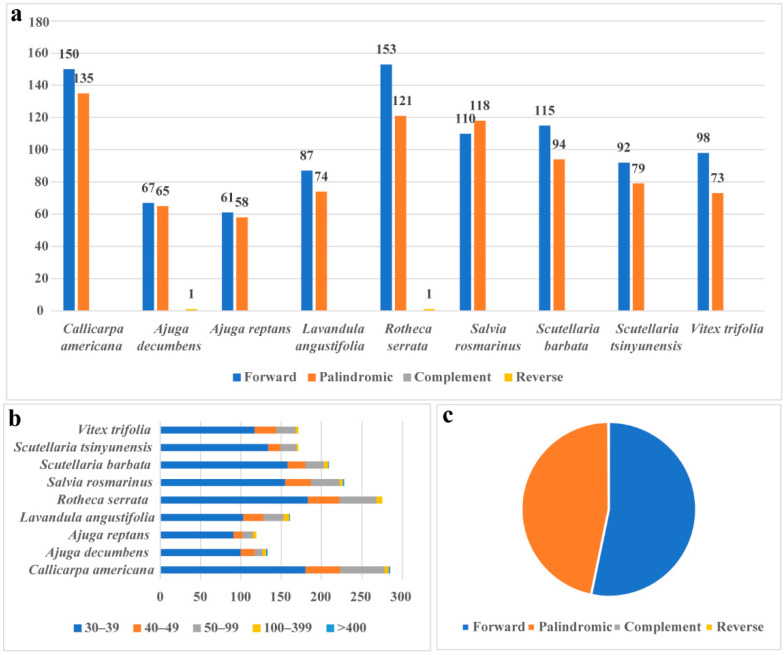
The types and existence of dispersed repetitive sequences in the mitochondrial genomes of 9 species of Lamiaceae. (**a**) Type and number of dispersed repeats. (**b**) The number of repetitions divided by length. Classification of all repeat sequences into five categories based on their length. (**c**) Percentage of four repetitive sequences.

The total numbers of the four types of dispersed repeats (forward, palindromic, complementary, and reverse repeats) ranged from 119 to 285 among the different species (Figure 2a, Table S4). All repeat sequences were divided into five categories according to their length: 30–39 bp, 40–49 bp, 50–99 bp, 100–399 bp, and ≥400 bp. In these categories, repeats with lengths of 30–39 bp were the most common, accounting for 69.69% of the total, while repeats with lengths of more than 400 bp were the least frequent, accounting for only 0.46% of the total (Figure 2b). The most common types of repetitive sequences were forward and palindromic repeats, accounting for 53.25% and 46.63% of the total length of repetitive sequences, respectively. Reverse repeats were found in *Ajuga decumbens* and *Rotheca serrata* (Table S4).

### 3.4. Codon Usage and RNA Editing Events

The PCGs in the mitochondrial genomes of the nine species of Lamiaceae were analyzed, and their codon usage frequency and relative synonymous codon usage frequency (RSCU) were calculated (Table S5). The number of codons in these genes ranged from 8359 in *Vitex trifolia* to 11,088 in *Salvia rosmarinus* (Table S5). RSCU > 1 indicates that the codon is preferentially used. A total of 63 synonymous codons were identified, excluding stop codons. Among these codons, 29 have RSCU values greater than 1, whereas the other 32 have RSCU values less than 1. Only the RSCU values of Trp (encoded by UGG) and Met (encoded by AUG) are equal to 1 (Table S5). Leu is the most frequently encoded amino acid in all mitochondrial PCGs, ranging from 10.56% to 11.33%, while Cys is the least frequently encoded, ranging from 1.40% to 1.62% (Table S5).

In the mitochondrial genome PCGs of the nine species of Lamiaceae, the number of RNA editing sites ranged from 369 to 494. A total of 494 RNA editing events were identified in mitochondrial PCGs of *C. americana* (Table S6). Among the mitochondrial PCGs of the nine Lamiaceae species, *nad4* showed the highest number of RNA editing events, with 41 to 46 identified editing sites. On the other hand, no RNA editing events were found in *atp1* (Table S6). In addition, we observed that changes at the second codon position accounted for the majority of changes in RNA editing sites in all nine Lamiaceae species, followed by changes at the first codon position. Most of the observed edits were conversions from Arg to Gly (13%), followed by Arg to Val (9.2%); only a small portion of Leu residues were converted into stop codons (1.6%) or Trp (1.6%) or Cys (1.2%) (Table S6). Pro to Leu is the most common proline mutation.

### 3.5. Intracellular Gene Transfer from Chloroplast to Mitochondrial Organelles

**Figure 3:**
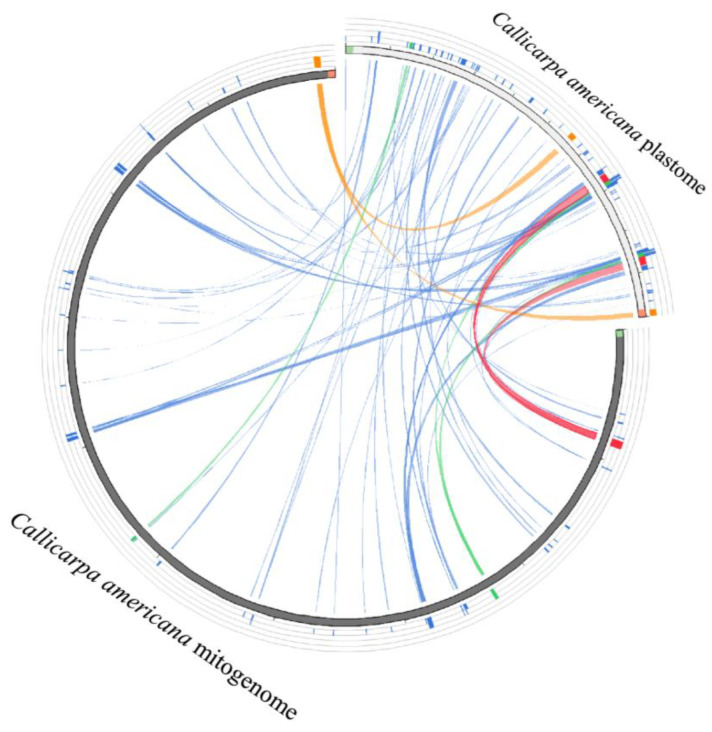
Homology analysis of mitochondrial and chloroplast genomes of *C. americana*. The colored blocks outside the sequence describe the explosion hit score, with the best quartile in red, followed by orange, green, and blue, respectively.

In the mitochondrial genome of *C. americana*, a total of 42 homologous fragments were identified that were shared with chloroplasts (excluding sequences aligned with chloroplast repeats) (Figure 3, Table S7). These fragments had migrated from chloroplasts to mitochondria as mitochondrial plastid DNA fragments (MTPTs). The length of homologous fragments identified in the *C. americana* mitochondrial genome ranged from 55 to 2596 bp. The total length of these MTPTs was 21,464 bp, accounting for about 4.30% of the entire mitochondrial genome (Table S7). These homologous sequences were annotated. Two protein-coding genes (*rpl23* and *rpl2*) and 11 tRNA genes (*tRNA-Asn* (GUU), *tRNA-Trp* (CCA), *tRNA-Pro* (UGG), *tRNA-Ile* (CAU and GAU), *tRNA-Ala* (UGC), *tRNA-Asp* (GUC), *tRNA-His* (GUG), *tRNA-Met* (CAU), and *tRNA-Ser* (GCU and GGA)) were identified in the mitochondrial genome of *C. americana*.

### 3.6. Genomic Characteristics of the Mitochondrial Genome in C. americana

**Figure 4:**
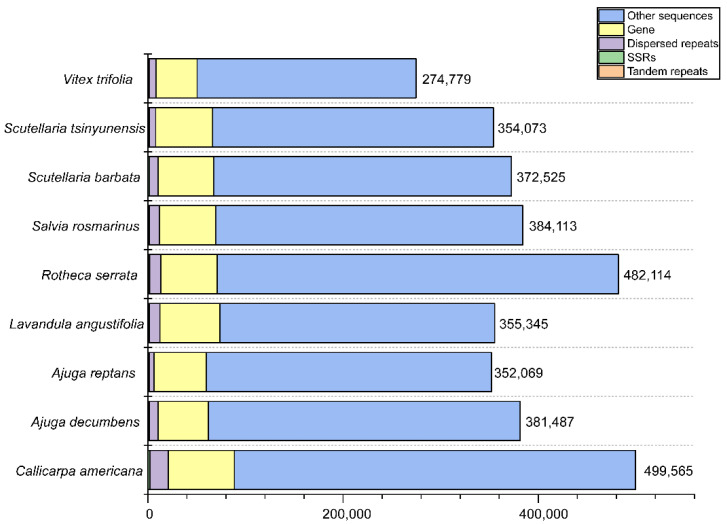
Genome size and content of the mitochondrial genomes of nine species of Lamiaceae. The figure shows the genome size of tandem repeats, SSRs, dispersed repeats, and gene coverage, and the proportion of each genome. The schematic tree below shows the phylogenetic relationships between species.

**Figure 5:**
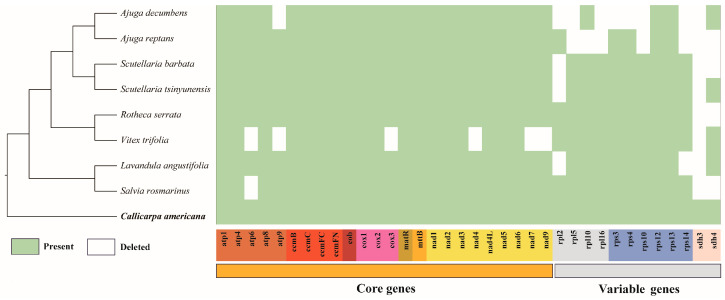
The distribution of PCGs in the mitochondrial genomes of *C*. *americana* and other Lamiaceae plants. The green box indicates that the gene exists, the light green box indicates that the gene is a pseudogene, and the white box indicates that the gene is missing in the mitochondrial genome.

During the long process of plant evolution, the size, GC content, and gene number of the mitochondrial genome have frequently changed. In Lamiaceae species (ranging from 274,779 bp to 499,565 bp), the difference in mitochondrial genome size is relatively large compared to Orobanchaceae species (ranging from 401,628 bp to 547,032 bp) (Table S1). The smallest mitochondrial genome in Lamiaceae is *V. trifolia*, with a size of 274,779 bp, while the largest is *C. americana*, with a size of 499,565 bp (Table S1). The number of complementary sequences and reverse sequences of all plants is very small and almost negligible. The number of forward sequences is usually close to or slightly lower than the number of palindrome sequences (Figure 4). The average GC content of Lamiaceae species (45.23%) was higher than that of Orobanchaceae species (44.01%). Specifically, the GC content in Lamiaceae species ranged from 44.77% to 45.62% (Table S1). The core and variable genes in the mitochondrial genome of Lamiaceae species have been lost to varying degrees (Figure 5). Among the Lamiaceae mitochondrial genomes, only *Ajuga reptans*, *C. americana*, *L. angustifolia*, *Rotheca serrata*, *Scutellaria barbata,* and *S. tsinyingensis* retained all core gene, whereas the variable genes of *A. decumbens* and *A. reptans* exhibited substantial loss (Figure 5).

### 3.7. Phylogenetic and Collinearity Analysis

**Figure 6:**
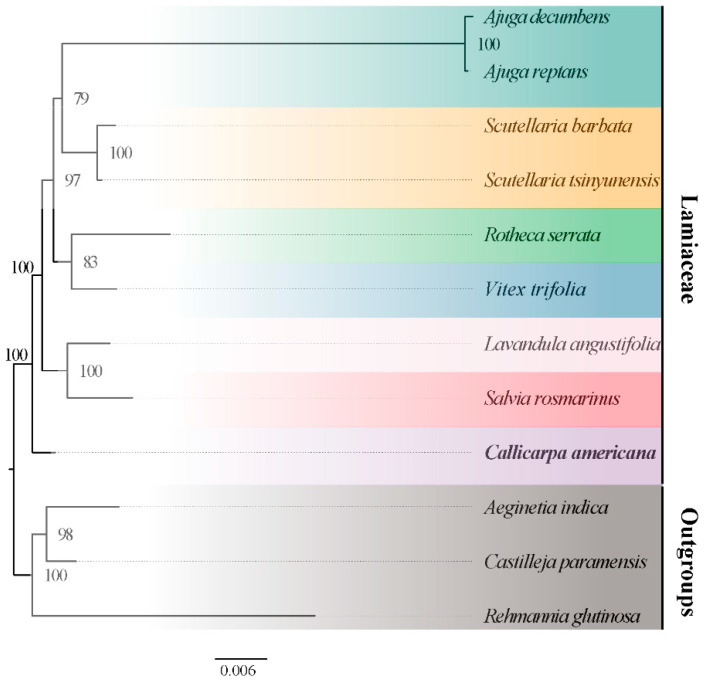
Maximum likelihood (ML) tree based on alignment of 32 PCG sequences of nine species of Lamiaceae. The numbers near the node represent different bootstrap percentages.

In order to determine the phylogenetic position of *Callicarpa americana* at the mitochondrial genome level, the mitochondrial genomes of the nine species of Lamiaceae were compared, and three species of Orobanchaceae were used as outgroups (Table S1, Figure 6). A phylogenetic tree was constructed based on the alignment of 32 PCG sequences (Figure 6). The results revealed that *C. americana* (*Callicarpa*) is a sister group to other Lamiaceae species; the support rate was 100%. The remaining Lamiaceae species, such as the two species of the *Ajuga*, are tightly clustered, indicating that they belong to the same family and are closely related. Similarly, the species in the other genera also formed close clusters. *Lavandula angustifolia* and *S. rosmarinus* formed a branch with a support rate of 100%, indicating a very close and highly reliable genetic relationship.

In the Lamiaceae ML tree, most of the relationships between genera are fully supported. The genetic relationship between *L. angustifolia* and *S. rosmarinus* received 100% node support. However, there are two nodes with low support, *A*. *reptans* was identified as a sister group to *R. serrata*, but this node received only 79% support (Figure 6). In general, except for the two low-support nodes mentioned above, other Lamiaceae genera have stable phylogenetic positions.

**Figure 7:**
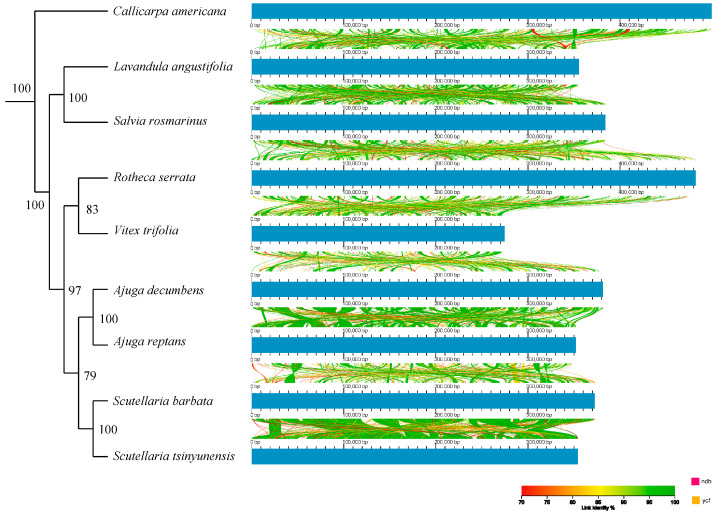
Covariance analyses of nine Lamiaceae species. Arcs from red to green indicate linkage identities between 70% and 100%. The schematic tree on the left shows the phylogenetic relationships between species.

In order to further study the collinearity of the mitochondrial genome of Lamiaceae, the mitochondrial genome structures of the nine Lamiaceae species were visualized using AliTV. The results showed that the gene homology was generally conserved in Lamiaceae (Figure 7). The mitochondrial genomes also contained many homologous collinearity fragments spanning most regions. However, the lengths of these homologous syntenic fragments varied, with homologous species with closer genetic relationships having longer homologous syntenic fragments (Figure 7).

## 4. Discussion

### 4.1. Mitogenome Architecture and Gene Content

Compared with the large number of plastid genomes that have been completely sequenced, reports of fully assembled plant mitochondrial genomes remain relatively scarce [41]. Although more than 13,000 complete plastid genomes are currently available in the NCBI database, completely resolved plant mitogenomes—and especially species with both organellar genomes sequenced—remain rare [41]. The *Callicarpa americana* mitogenome assembled in this research exhibits a multi-branched yet closed-circular configuration maintained by a high density of repetitive elements. The final assembly comprises ten contigs that collectively form a 499,565 bp mitogenome, representing the largest mitogenome reported thus far within Lamiaceae.

The GC content of *C. americana* (45.3%) is comparable to that of other Lamiaceae mitogenomes, such as *Ajuga reptans* (47.6%) [42], *Scutellaria tsinyunensis* (45.26%) [43], and *Thymus mongolicus* (45.6%) [44], all falling within the conservative range of 45–50% typically observed across angiosperms. The genome encodes a total of 63 genes, including 37 protein-coding genes (PCGs), 23 tRNA genes, and 4 rRNA genes. This gene content and composition is consistent with most land-plant mitogenomes [45]. The conserved core gene set includes ATP-synthase (*atp1*–*atp9*), NADH-dehydrogenase (*nad1*–*nad9*), cytochrome oxidase (*cox1*–*cox3*), cytochrome c biogenesis (*ccmB*, *ccmC*, *ccmFC*, *ccmFN*), and ribosomal protein genes from both large and small subunits, reflecting the functional stability of the oxidative phosphorylation machinery within Lamiaceae mitochondria [46].

The mitochondrial genome of *C. americana* (499.6 kb) is substantially larger than that of *Prunella vulgaris* (297.8 kb) [14], one of the smallest reported in the family. Such variation is predominantly attributable to differences in non-coding regions, repeat proliferation, and inter-organellar sequence insertions rather than to changes in gene number. Structurally, the mitogenome of *C. americana* conforms to the typical circular master structure of most angiosperms [47], although dynamic conformational isomers likely coexist due to repeat-mediated homologous recombination. Multi-chromosomal organizations have also been reported in other Lamiaceae members; for instance, *Salvia officinalis* possesses two circular chromosomes [48].

In *C. americana*, homologous recombination, mediated by two pairs of long repeats (LR3 and LR5), generates alternative genome configurations, supporting the view that plant mitogenomes exist as a population of interconvertible molecules rather than a single static circle. The unequal representation of recombination products—for example, 28.22% versus 71.78% for different LR-mediated configurations—suggests that recombination frequency is under cellular regulation rather than occurring as random stochastic events [49]. Similar mechanisms have been documented in *Oryza sativa* [50], where recombination among repeated elements maintains structural diversity and genome equilibrium.

### 4.2. Repetitive Elements and Genome Complexity

Repetitive elements are key drivers of structural plasticity and evolutionary expansion in plant mitochondrial genomes [51]. In the *C. americana* mitogenome, a large number of simple sequence repeats (SSRs), tandem repeats, and dispersed repeats were identified, collectively contributing to its considerable genome size (499,565 bp). Among the nine Lamiaceae species examined, the total number of SSRs ranged from 71 to 125 per species, with *C. americana* exhibiting a relatively high abundance. Tetranucleotide repeats represented the dominant motif, accounting for 35.89% of all SSRs, followed by dinucleotide (15.35%) and mononucleotide (11.26%) repeats. This predominance of tetranucleotide motifs aligns with previous findings in *Lithocarpus litseifolius* (32.57%) [52], indicating that such motifs are preferentially maintained in certain lineages, whereas other plant groups such as *Hedychium* membranaceus exhibit different SSR composition patterns [53,54].

Tandem repeats ranging from 212 to 539 bp were detected throughout the *C. americana* mitochondrial genome. This is similar to the repeat length distribution observed in other Lamiaceae species [15,48] (Table S3). Dispersed repeats were also abundant, with total counts varying between 119 and 285 across species (Table S4). In *C. americana*, forward and palindromic repeats constituted the majority—53.25% and 46.63% of all dispersed repeats, respectively—whereas reverse and complementary repeats were rare. Most dispersed repeats fell into the 30–39 bp length category (69.69%), while only 0.46% exceeded 400 bp. These length- and type-specific distributions indicate that short, AT-rich repeat sequences are especially prone to replication slippage and recombination, thereby driving mitogenome instability and contributing to genome expansion. This tendency is consistent with previous findings that nucleotide composition strongly influences the formation and variability of simple sequence repeats [55].

The predominance of AT-rich SSRs and the high proportion of short forward and palindromic repeats observed here are consistent with the findings reported in *S. miltiorrhiza* [15]. These AT-rich sequences exhibit reduced thermodynamic stability, increasing their susceptibility to strand slippage and illegitimate recombination during replication. As a result, they likely promote rapid structural diversification of the *C. americana* mitogenome and contribute to the expansion of its non-coding regions [56].

### 4.3. Codon Usage Patterns and Translation Optimization

Codon usage bias is a key genomic feature that reflects both mutational tendencies and natural selection acting on translational efficiency, accuracy, and metabolic optimization in plant mitochondria [57]. In the mitogenome of *C. americana*, a total of 63 sense codons (excluding stop codons) were identified across 37 protein-coding genes—a number comparable to those in other Lamiaceae species such as *Vitex trifolia* and *Salvia rosmarinus* (8359–11,088 codons) (Table S5). Among these, 29 codons exhibited relative synonymous codon usage (RSCU) values greater than 1, while 32 had RSCU values below 1, suggesting a weak yet consistent codon usage bias characteristic of angiosperm mitogenomes. The UGG codon (Trp) and AUG codon (Met) displayed neutral usage (RSCU = 1), indicating balanced selection for these essential residues. Leucine was the most frequently encoded amino acid, representing approximately 10.56–11.33% of all codons, while cysteine was the least frequently encoded (1.40–1.62%). This amino acid usage pattern aligns with the mitogenomes of *Lavandula angustifolia* [16] and other Lamiaceae species, underscoring the evolutionary conservation of translational preference across the family. The overall bias toward codons ending in A or U indicates a mutational pressure favoring AT-rich sequences, consistent with the mitogenome’s GC content (44.8–45.6%) and the tendencies observed in *Leonurus japonicus*, *V. rotundifolia*, and other species of Lamiaceae [12]. Such AT-enrichment may arise from replication-associated deamination or biased repair mechanisms that gradually shape mitochondrial codon composition [58].

In addition to mutation pressure, translational selection likely contributes to codon preference in *C. americana*. Codons corresponding to abundant mitochondrial tRNAs are used more frequently, reducing translational time and enhancing protein synthesis efficiency. This co-adaptation between codon usage and tRNA availability represents an important mechanism for maintaining translational accuracy and energy efficiency in mitochondria [59]. Moreover, the relatively uniform RSCU distribution across the Lamiaceae family indicates that selection acts to preserve translational stability despite structural genome variability. Together, these observations suggest that both mutational and selective forces drive codon usage evolution in the *C. americana* mitogenome. The predominance of A/U-ending codons and the enrichment of leucine and serine residues indicate a weak but consistent directional bias, suggesting a balance between genome compositional constraints and translational optimization. This codon usage balance, which is also evident in other Lamiaceae species [12,43,44], suggests a conserved evolutionary strategy that supports stable mitochondrial gene expression and maintains metabolic functionality across the family.

### 4.4. RNA Editing Site Prediction and Functional Implications

RNA editing represents a vital post-transcriptional regulatory process that enhances transcript diversity and protein functionality in plant mitochondria. It primarily involves site-specific cytidine (C)-to-uridine (U) conversions that often restore conserved amino acid residues lost during DNA-level mutations [52]. In the *C. americana* mitogenome, a total of 494 C-to-U editing sites were identified across 37 protein-coding genes (Table S6), which ranked highest among the numbers recorded within Lamiaceae. This finding suggests a highly active RNA editing system that may compensate for genomic mutational drift by restoring evolutionarily conserved codons. Editing events in *C. americana* were predominantly concentrated in the first and second codon positions, which is consistent with observations in other angiosperms such as *Leonurus japonicus* and *Salvia miltiorrhiza* [12,15]. These positions are most likely to induce nonsynonymous substitutions, thereby altering amino acid sequences and modulating protein structure and function. The most frequent amino acid conversions included Arg → Gly (13%) and Arg → Val (9.2%), while rare edits produced stop codons from Leu (1.6%) or converted Leu to Trp and Cys (each 1.6–1.2%) (Table S6). Such amino acid changes have been shown to increase local hydrophobicity or promote tighter packing within transmembrane helices, thereby enhancing the conformational stability of membrane-embedded respiratory enzyme complexes, as supported by previous studies.

Gene-specific analysis revealed that the *nad4* gene harbored the largest number of editing sites (41–46), while *atp1* and *sdh3* lacked any detectable editing. This heterogeneity indicates functional differentiation among mitochondrial genes, with those involved in electron transport and energy metabolism existing under stronger post-transcriptional regulation. Similar gene-specific editing patterns have been observed in *Ajuga decumbens* and *Physcomitrella patens*, supporting the evolutionary conservation of editing hotspots within plant mitogenomes [42,60]. The extensive RNA editing observed in *C. americana* likely contributes to post-transcriptional optimization of mitochondrial proteins, thereby supporting stable organellar function. The elevated editing frequency may also represent an adaptive mechanism that helps maintain efficient respiratory activity in the presence of structural genome rearrangements and an AT-rich sequence background.

### 4.5. Inter-Organellar DNA Transfer and Genomic Evolution

Intracellular DNA transfer is a hallmark of plant organelle genome evolution, reflecting the ongoing exchange of genetic material among the chloroplast, mitochondrion, and nucleus [61]. In the *C. americana* mitogenome, a total of 42 chloroplast-derived mitochondrial plastid DNA fragments (MTPTs) were identified, accounting for 21,464 bp, approximately 4.30% of the entire genome (Figure 3, Table S7). These transferred sequences ranged from 55 bp to 2596 bp and included two intact protein-coding genes (*rpl23*, *rpl2*), as well as 11 tRNA genes (*trnAsn-GUU*, *trnTrp-CCA*, *trnPro-UGG*, *trnIle-CAU/GAU*, *trnAla-UGC*, *trnAsp-GUC*, *trnHis-GUG*, *trnMet-CAU*, *trnSer-GCU/GGA*). The relatively high proportion of MTPTs in *C. americana* compared with other Lamiaceae members indicates active historical gene exchange between organelles and suggests that DNA transfer is an important contributor to genome expansion within this family. The prevalence of MTPTs in *C. americana* (4.30%) exceeds that reported for several other angiosperms such as *Acer truncatum* (2.36%) [62] and *Salix suchowensis* (2.8%) [63], but is consistent with high-transfer lineages including Fabaceae and Poaceae. Such differences likely result from lineage-specific variations in organelle dynamics, repeat content, and recombination frequency. The presence of intact chloroplast genes and functional tRNAs within the *C. americana* mitochondrion implies that some transferred fragments may remain transcriptionally active and contribute to organellar metabolism. Similar functional insertions have been reported in *Lavandula angustifolia* [16] and *Salvia miltiorrhiza* [15], suggesting that MTPTs can occasionally acquire regulatory or structural roles rather than acting solely as non-coding relics.

Mechanistically, repeat-mediated recombination is believed to facilitate the integration of plastid fragments into mitochondrial DNA. The dispersed repeat-rich regions of the *C. americana* mitogenome likely provide homologous anchors for recombination, enabling chloroplast sequences to be captured during replication or repair events [64]. Once incorporated, these fragments tend to cluster in intergenic or intronic regions, contributing to genome enlargement and architectural complexity. Over time, neutral or beneficial fragments may be selectively retained, while deleterious ones are eliminated through recombination or gene conversion. From an evolutionary perspective, MTPT accumulation enhances the “genomic mosaic” character of plant mitochondria [65]. The coexistence of native mitochondrial genes and chloroplast-derived sequences generates hybrid regulatory landscapes, blurring organelle-specific evolutionary histories. Such chimeric architectures complicate phylogenetic inference but provide a valuable resource for functional innovation and adaptive evolution. In *C. americana*, the high level of cp-to-mt DNA transfer underscores an active genomic interplay between organelles, promoting plasticity in both structure and function.

### 4.6. Phylogenetic Analysis and Genome Structural Evolution

Phylogenetic analysis of mitochondrial genomes provides critical insights into evolutionary relationships and structural diversification across Lamiaceae. Using 38 conserved PCGs from nine representative Lamiaceae species and three Orobanchaceae outgroups, a maximum likelihood (ML) phylogenetic tree was constructed (Figure 6). The results indicate that *C. americana* is a sister group to the other species of Lamiaceae, with 100% bootstrap support, indicating a close genetic relationship and confirming the placement of *Callicarpa* within the basal lineage of the subfamily Callicarpoideae. This finding is consistent with plastome-based classifications of Lamiaceae [9] and further validates the phylogenetic reliability of mitochondrial genomes for resolving deep evolutionary nodes within this family.

The other Lamiaceae species displayed distinct clustering patterns corresponding to their respective genera. The two *Ajuga* species clustered together, while *Scutellaria barbata* and *S. tsinyunensis* also formed a monophyletic group, reflecting strong intrageneric genetic cohesion. However, two nodes exhibited slightly lower support—the groupings of *Ajuga reptans* with *Rotheca serrata* (79%) and *Pogostemon heyneanus* with *Scutellaria barbata*—suggesting potential lineage-specific rearrangements or horizontal transfer events that obscure strict vertical inheritance. The low support for the phylogenetic relationship may also be due to the insufficient informative sites provided by mitochondrial coding genes. Similar topological ambiguities have been reported in the chloroplast phylogenies of Lamiaceae, where frequent recombination and gene loss introduce minor tree topology instability [9,66].

Synteny analysis using AliTV revealed that the mitochondrial genomes of Lamiaceae retain extensive collinearity across major regions, though numerous rearrangements and inversions were observed among distantly related genera (Figure 7). Closely related species, such as *Scutellaria barbata* and *S. tsinyunensis*, exhibited long conserved collinear blocks, whereas intergeneric comparisons—such as *Callicarpa* versus *Ajuga*—displayed pronounced structural fragmentation. This pattern highlights a dual evolutionary trend: conservation of gene order within genera and substantial reorganization between lineages. Similar rearrangement patterns have been documented in *Thymus mongolicus* and *Phlomoides rotata*, suggesting that repeat-mediated recombination is a primary driver of mitochondrial structural evolution in Lamiaceae.

## 5. Conclusions

In this study, the complete mitochondrial genome of *Callicarpa americana* was successfully assembled for the first time, revealing the unique configuration in its mitochondrial genome structure. The mitochondrial genome of *C. americana* has a multi-branched structure with a total length of 499,565 bp. In addition, the genome has 64 genes, including 37 protein-coding genes, 23 tRNA genes, and 4 rRNA genes. The research presented here indicates that the main factor affecting mitochondrial genome size in Lamiaceae is scattered repetition. Moreover, chloroplast sequence transfer accounted for 4.30% of the mitochondrial genome of *C. americana*, and the insertion of MTPTs was not related to mitochondrial genome size. The phylogenetic tree constructed using 32 aligned protein-coding genes revealed that *Callicarpa* is positioned as a sister group to the other members of Lamiaceae. This work complements previously published information regarding the mitochondrial genomes of the Lamiaceae family and provides a reference for their future evolutionary studies.

## Figures and Tables

**Table 1:** The location length and depth of each assembled contig in *C*. *americana*.

Sequences	Start	End	Length (bp)	Depth
Contig1	1	247,270	247,270	33.8x
Contig2	247,271	288,085	40,815	33.7x
LR3	288,086	290,249	2164	64.9x
	497,402	499,565		
Contig4	290,250	343,113	52,864	28.7x
LR5	343,114	348,045	4932	61.7x
	446,893	451,824		
Contig6	348,046	355,794	7749	31.3x
Contig7	355,795	367,136	11,342	37.9x
Contig8	367,137	446,892	79,756	35.2x
Contig9	451,825	497,401	45,577	31.6x

Note: LR stands for long repeat sequences.

**Table 2:** *C. americana* mitogenome annotation results.

Group of Genes	Gene Content
ATP synthase	*atp1* (2), *atp4*, *atp6*, *atp8*, *atp9*
Cytochrome c biogenesis	*ccmB*, *ccmC*, *ccmFC* *, *ccmFN*
Ubichinol cytochrome c reductase	*cob*
Cytochrome c oxidase	*cox1*, *cox2* *, *cox3*
Maturases	*matR*
Transport membrane protein	*mttB*
NADH dehydrogenase	*nad1* ****, *nad2* ****, *nad3*, *nad4* ***, *nad4L*, *nad5* ****, *nad6*, *nad7* ***, *na d9*
Ribosomal proteins (LSU, large subunit)	*rpl10*, *rpl16*, *rpl2*, *rpl5*
Ribosomal proteins (SSU, small subunit)	*rps10* *, *rps12*, *rps13*, *rps14*, rps3*, *rps4*
Succinate dehydrogenase	*sdh3*, *sdh4*
Ribosomal RNAs	*rrn18*, *rrn26* (2), *rrn5*
Transfer RNAs	*trnF-GAA* (2), *trnL-CAA*, *trnC-GCA*, *trnD-GUC*, *trnE-UUC*, *trnG-GCC*, *trnH-GUG*, *trnK-UUU*, *trnI-CAU*, *trnM-CAU*, *trnfM-CAU* (2), *trnN-GUU*, *trnP-UGG*, *trnQ-UUG*, *trnS-GCU*, *trnS-GGA*, *trnS-UGA*, *trnW-CCA*, *trnY-GUA*, *trnA-UGC, trnP-CGG*

Note: * Gene that contains one intron; *** Gene that contains three introns; **** Gene that contains four introns; Gene (2) that contains two copies.

**Table 3:** The number and proportion of recombinant molecules mediated by repeated sequences of *C. americana* mitogenomes.

Repeat	Length (bp)	Reads Span Across Regions	Reads Support	Total Reads Support
LR3	2164	Contig1-LR3-Contig4	1652	5071 (40.02%)
		Contig2-LR3-Contig9	1761	
		Contig1-LR2-Contig3	1873	3413 (59.98%)
		Contig3-LR4-Contig9	3198	
LR5	4932	Contig4-LR5-Contig8	6412	10,635 (68.13%)
		Contig6-LR5-Contig9	4223	
		Contig4-LR5-Contig9	2563	4974 (31.87%)
		Contig6-LR5-Contig8	2411	

## Data Availability

The mitochondrial genome of *Callicarpa americana* (PX121882) is publicly available in Genbank at NCBI.

## Supplementary Materials

The following supporting information can be downloaded at: https://www.mdpi.com/article/10.3390/biology14121747/s1, Table S1: The GenBank accession numbers and information on other features of the mitogenomes used in this study; Table S2: Types and numbers of SSRs in the nine Lamiaceae mitogenomes; Table S3: The number and total length of tandem repeats in the nine Lamiaceae mitogenomes; Table S4: Types and numbers of dispersed repeats in the nine Lamiaceae mitogenomes; Table S5: Codon usage within the PCGs of the nine Lamiaceae mitogenomes; Table S6: The RNA editing sites identified in *Callicarpa americana* mitogenome; Table S7: The identification MTPTs in *Callicarpa americana* mitogenome.

## Abbreviations

The following abbreviations are used in this manuscript:

CMS	Cytoplasmic male sterility
MTPT	Mitochondrial-to-plastid DNA transfer
NCBI	National Center for Biotechnology and Information
SSRs	Simple Sequence Repeats
RSCU	Relative synonymous codon usage
